# Cellular Players in the Herpes Simplex Virus Dependent Apoptosis Balancing Act

**DOI:** 10.3390/v1030965

**Published:** 2009-11-18

**Authors:** Marie L. Nguyen, John A. Blaho

**Affiliations:** 1 Department of Microbiology and Immunology, Des Moines University, Des Moines, IA, USA; 2 Department of Microbiology, Mount Sinai School of Medicine, New York, NY, USA; E-Mail: john.blaho@mssm.edu

**Keywords:** Herpes Simplex Virus, apoptosis, virus-host interactions

## Abstract

Apoptosis is triggered as an intrinsic defense against numerous viral infections. Almost every virus encodes apoptotic modulators, and the herpes simplex viruses (HSV) are no exception. During HSV infection, there is an intricate balance between pro- and anti-apoptotic factors that delays apoptotic death until the virus has replicated. Perturbations in the apoptotic balance can cause premature cell death and have the potential to dramatically alter the outcome of infection. Recently, certain cellular genes have been shown to regulate sensitivity to HSV-dependent apoptosis. This review summarizes current knowledge of the cellular genes that impact the apoptotic balance during HSV infection.

## 1. Introduction

Apoptosis is a form of programmed cell death that is triggered during normal development and as a response to cellular stresses. Apoptotic cells display unique biochemical and morphological changes that distinguish them from cells dying through other pathways. Apoptotic morphological characteristics include membrane blebbing, chromatin condensation, nuclear fragmentation, and exposure of phosphatidylserine moieties [1–3]. There are two well defined pathways for the induction of apoptosis [4,5]. In one pathway, the extrinsic pathway, the signal is initiated at the cell surface [6]. Here, death factors bind to receptors embedded in the plasma membrane. The ligand:receptor binding triggers the assembly of large multiprotein complexes called death inducing signaling complexes (DISCs). A key biochemical characteristic of classical apoptosis is the activation of a specific set of cysteinyl aspartate proteases, called caspases. DISC formation includes the recruitment of the initiator caspase 8 to the complex that causes its autoactivation. Active caspase 8 cleaves and activates downstream effector caspases, such as caspases 3 and 7. Effector caspases cleave cellular proteins which maintain the integrity of the cell, which leads to the biochemical and morphological changes associated with apoptosis.

The other major signaling pathway leading to apoptosis is the intrinsic pathway [7]. Intrinsic triggers of apoptosis include DNA damage, heat-shock, and damage due to reactive oxygen species. This response causes changes in the ratio of pro- and antiapoptotic Bcl-2 family members within the mitochondrial membrane, which leads to a release of cytochrome c into the cytoplasm [8,9]. Cytochrome c forms complexes with apaf-1 and procaspase 9 to form a large multicomponent structure known as the apoptosome, causing the activation of caspase 9 [5,8]. Active caspase 9 goes on to activate effector caspases and the apoptosis inducing signaling pathways converge at this point. An additional layer of complexity is added to these apoptotic signaling pathways as crosstalk between the two pathways exists. For example, caspase 8 activated during the extrinsic pathway cleaves the Bid Bcl-2 family member [5]. The cleaved product, tBid, acts as a positive regulator of mitochondrial cytochrome c release. This release, in turn, initiates the intrinsic apoptotic pathway and amplifies the extrinsic apoptotic death signal.

The apoptotic pathway is often triggered as a consequence of viral infection. Therefore, almost all viruses encode genes to modulate the apoptotic pathway. It is important to note that although DNA viruses seem to universally express anti-apoptotic proteins, RNA viruses rarely do [10]. One potential explanation for this observation is that the ability of RNA viruses to replicate rapidly may allow them to avoid the problems associated with apoptosis. [11]. DNA viruses use a diverse array of mechanisms to impair the apoptotic response of cells. Small DNA tumor viruses, like human papillomavirus and adenovirus block the effects of p53 that normally functions to upregulate multiple pro-apoptotic cellular genes during times of cell stress [12–14]. Insect viruses, such as Baculovirus, produce proteins which inhibit caspases [15]. A subset of herpesviruses, including Kaposi’s sarcoma herpes virus / human herpes virus 8 and Epstein Barr virus encode genes that are homologous to cellular anti-apoptotic Bcl-2 family members [16–18]. Herpes simplex viruses (HSVs) encode a number of genes that modulate cellular apoptotic pathways.

HSV infection triggers the apoptotic pathway early in infection [19–22]. However, as the viral infection progresses, anti-apoptotic proteins produced by the early and late herpes viral genes block apoptosis from ensuing [19,23,24]. This sets up an intricate balance between the pro- and anti-apoptotic factors in the cell. When the anti-apoptotic factors are not efficiently produced, the balance is upset and the HSV-infected cells die through an apoptotic pathway, Herpes Simplex Virus-Dependent Apoptosis (HDAP) [19,25,26]. Initial research on HDAP focused on the viral factors modulating apoptosis during infection. These studies identified a number of viral proteins which act to block apoptosis during infection. They include immediate early genes, such as ICP27 and ICP4, that likely act as upstream regulators of later anti-apoptotic viral genes [19,25,26]. Deletion of either of these genes from the viral genome results in a replication defective and pro-apoptotic virus. The early gene, ICP10 PK, plays an anti-apoptotic role during HSV-2 infection [27,28]. Late viral genes shown to possess anti-apoptotic properties include Us3, gD, gJ, and the latency associated transcripts [29–33]. Single deletions of these late genes generate viruses that fail to cause apoptosis to the same extent as ICP27 and ICP4-null viruses, suggesting that the late viral genes act in concert to prevent apoptosis during a wild type HSV infection.

HSV is best known as the causative agent of cold sores and genital herpes. However, when the virus reaches tissues other than the mucosal epithelium, it causes much more serious disease such as potentially blinding cases of keratitis and life threatening encephalitis. Animal and human studies have provided support for a role for apoptosis in the pathogenesis severe HSV disease. Specifically, apoptotic cells have been detected in animal models of herpes simplex keratitis [34–36]. The amount of apoptosis inversely correlates with the severity of disease [37,38]. This result suggests that apoptosis is acting as an antiviral defense mechanism of infected cells. Apoptosis has also been found to be a feature of herpes simplex encephalitis (HSE) [28,39,40]. In this case, however, there seems to be a positive correlation between the severity of disease and extent of apoptosis [40,41]. Thus, triggering apoptosis may contribute to the virulence of HSV during encephalitis. Together, the evidence suggests that cell-type determinants mediate the way in which apoptosis impacts HSV pathogenesis.

## 2. Results and Discussion

The majority of the early studies on HDAP utilized immortalized cell lines typically used to propagate HSV, *i.e.*, Vero and HEp-2/HeLa cells, which provided insight into the viral factors involved in this response. Yet, later studies demonstrated the existence of cell-type differences in the response to HDAP, indicating that cellular factors play an important role in this process. For example, primary epithelial cells from mammary tissues and primary skin fibroblasts display resistance to HDAP [42,43]. Primary murine neuronal cells also seem to show a similar apoptotic regulation during wild type HSV-1 infection [44–46]. Recently, a number of specific cellular factors modulating HDAP have been identified. These cellular genes can be classified as caspases, Bcl-2 family members, NF-κB, and oncogenic genes.

### 2.1. Caspases

Multiple studies demonstrate that HDAP is caspase-dependent [19,23,47–50]. The small peptide pan-caspase inhibitors, e.g., *z*-VAD-fmk, are efficient suppressors of the biochemical and morphological apoptotic phenotypes found during HDAP. The use of specific caspase inhibitors has allowed elucidation of the pathway by which apoptosis is triggered during infection [47]. An inhibitor of caspase 9, *z*-LEHD-fmk, suppressed HDAP in HEp-2 cells to an extent similar to that of the pan-caspase inhibitor. However, a caspase 8 inhibitor, *z*-LETD-fmk failed to suppress HDAP, even though it was capable of suppressing apoptosis induced by tumor necrosis factor and cycloheximide. Furthermore, cytochrome c was released in a caspase-independent manner during HDAP [47]. These results led to the conclusion that HDAP occurs through the intrinsic pathway of apoptosis.

One effector caspase commonly utilized in the intrinsic pathway is caspase 3. Studies in our laboratory using genetic analysis demonstrated that caspase 3 is necessary for HDAP. This work stemmed from our observation that the tumor cells that were resistant to HDAP were also highly resistant to other forms of apoptotic induction. We hypothesized that the HDAP resistance was due to the acquisition of mutations in cellular apoptotic pathways, which blocked apoptosis in general [43]. One such tumor cell line was the breast cancer MCF-7 cells. MCF-7 cells are known to be defective for a key component of the apoptotic cascade, caspase 3 [51]. When MCF-7 cells in which caspase 3 expression was restored (MCF-7 C3) and caspase 3-null counterparts (MCF-7 PV) were infected with a pro-apoptotic HSV mutant, the MCF-7 C3 cells displayed membrane blebbing and biochemical features of apoptosis, while similarly infected MCF-7 PV cells did not [49]. This finding not only demonstrated that restoring the apoptotic pathway sensitized these cells to HDAP, but also identified caspase 3 as a cellular determinant for HDAP. Interestingly, caspase 3 has also been found to play a role in the apoptosis during influenza virus infection [52]. Inhibition of caspase 3 leads to a reduction in the influenza virus titer. A subset of caspase 3 is found in the active form during a wild type HSV infection [49]. Whether caspase 3 similarly contributes to HSV replication efficiency has yet to be determined.

It is of note that the ICP22 protein of HSV has been shown to be cleaved by caspases during infection with a mutant, pro-apoptotic virus [53]. The biological significance of this cleavage has yet to be elucidated, as the cleaved form of the protein does not accumulate during a wild type HSV-1 infection, and the function of the cleaved form has not been defined.

### 2.2. Bcl-2 Family Members

The Bcl-2 family comprises of pro-and anti-apoptotic proteins all sharing conserved protein motifs, known as Bcl-2 homology (BH) domains [54]. Most of these members reside within the mitochondrial membrane. The ratio and activities of the pro- and anti apoptotic Bcl-2 family members in this region determines the release of cytochrome c from the mitochondria and subsequent initiation of the intrinsic apoptotic pathway. HSV has been shown to block the activities of certain pro-apoptotic Bcl-2 family members. HSV Us3 blocked cell death and caspase activation induced by overexpression of Bax, Bad, or Bid [55,56]. In fact, Bax appears to accumulate at mitochondria during HDAP [47]. Cartier *et al.* determined that Bad is phosphorylated in a Us3 dependent manner during HSV infection [57]. This may contribute to its ability to block Bad activity. In other studies, exogenous expression of the anti-apoptotic Bcl-2 protein blocked apoptosis induced by HSV infection of HEp-2 and the U-937 lymphoma cell line [48,58]. In U-937 cells this Bcl-2 overexpression led to increased HSV-2 yields, suggesting that manipulation of apoptotic pathways can influence the efficiency of virus replication, at least in certain cell types [58].

### 2.3. NF-κB

NF-κB is a transcription factor that plays a major role in inflammation and the immune response [59]. Inactive NF-κB is bound to an inhibitory protein, IκB, and sequestered in the cytoplasm. Following exposure to a proper stimulus, IκB is phosphorylated and degraded, and NF-κB translocates to the nucleus [60,61]. There, it induces the expression of a number of genes involved in cell survival, proliferation, and inflammation [62,63]. Early studies demonstrated that NF-κB translocates to the nucleus during HSV-1 infection and the timing of this translocation coincides with apoptosis prevention [64–67]. A pro-apoptotic mutant virus of HSV-1 did not cause a similar translocation [65]. Furthermore, cells which expressed a dominant negative form of IκBα, IκBαDN, display apoptosis when infected with wild type HSV [65–67]. These results support a role for NF-κB in the prevention of apoptosis during HSV-1 infection. However, others have reported that NF-κB may provide important functions other than apoptosis prevention during HSV-1 infection [68,69]. For example, studies suggested that NFκ-B activation is linked to the activation of a key regulator of the innate immune response, PKR [68,69].

More recently, studies from our group have provided additional information regarding a role for NF-κB in apoptosis prevention. HEp-2 cells infected with HSV-2 were found to display NF-κB nuclear translocation during the time of apoptosis prevention [70]. This result suggested that NF-κB’s role in the prevention of apoptosis during infection with HSV-2 is similar to that of its role in HSV-1 infection. Additionally, HSV infection led to NF-κB translocation in corneal epithelial cells, implicating NF-κB in apoptosis prevention in herpes simplex keratitis [71]. However, not all of HSV’s capacity to block apoptosis can be attributed to NF-κB. Results from our group have demonstrated that HSV-1 is effective at blocking apoptosis in HEp-2 cells induced by Fas ligand and cycloheximide [72]. Although this prevention corresponded with NF-κB nuclear translocation, HSV-1 was still capable of blocking Fas-mediated apoptosis in HEp-2 cells constitutively expressing IκBαDN [72]. This result led to the conclusion that HSV-1 possesses NF-κB-independent mechanisms of blocking apoptosis.

### 2.4. Oncogenic Genes

Oncogenes were first implicated as mediators of HDAP in studies comparing the sensitivities of human cancer cells with that of primary cells [42]. The studies indicated that the transformation status of cells correlates with their sensitivity to the viral induced apoptosis. Subsequently, differences between the immortalized, but not transformed Vero cell line and that of the highly sensitive transformed HEp-2 cells revealed the existence of a cellular protein that facilitates apoptosis during infection [73]. The Vero cells require production of the facilitator protein, while HEp-2 cells are independent of it. All primary cells tested to date have been resistant to HDAP based upon caspase substrate cleavage, chromatin condensation, membrane blebbing, and DNA laddering analyses [43]. Remarkably, even cells of the same patient derived from a mammary tumor and the surrounding normal tissue displayed opposite sensitivity to HDAP [43]. These data led to the hypothesis that genetic changes occurring during tumor development sensitized cells to HDAP.

Our group tested the aforementioned hypothesis using a tumor model system in which the cellular transformation status could be experimentally manipulated. This model makes use of the fact that the human cervical cancer derived HeLa cell line requires continuous expression of human papillomavirus (HPV) genes in order to maintain a tumorigenic phenotype [74–76]. Suppressing the HPV 18 E6 and E7 via the expression of the papillomavirus E2 transcriptional repressor leads to reductions in cell growth, and eventually senescence of the cells [74,77–79]. We found that repression of HPV E6 and E7 reduced the sensitivity of HeLa cells to HDAP [80]. Furthermore, adding back expression of HPV E6, but not E7, restored HeLa cell HDAP sensitivity. These studies allowed us to narrow our search for oncogenic HDAP mediators to those altered by HPV E6. HPV E6 is a multifunctional protein, known to affect the function of at least a dozen cellular proteins [81]. So far, two of these proteins, p53 and telomerase, have been identified as regulators of HDAP sensitivity.

#### 2.4.1. p53

HPV E6’s most well studied function is the inactivation of the cellular tumor suppressor, p53. The p53 protein has been dubbed the “guardian of the genome” due to its role in preventing genetic mutations in the cell lineage [82]. The levels of p53 protein are usually kept low in healthy cells due to an extremely rapid turnover mediated by mdm2. However in response to cell stresses, p53 is stabilized, accumulates within the cell, and forms tetramers. The tetramers of p53 act as a transcriptional factor to induce a variety of genes that are involved in DNA repair, cell cycle arrest, and apoptosis. HPV E6 forms complexes with p53 and the cellular ubiquitin ligase, E6AP/UBE3A, to cause p53 ubiquitination and degradation [83,84]. Inactivation of p53 can also be achieved by expressing a mutant p53 molecule, which oligomerizes with wild type p53, but lacks the ability for transcriptional activation [85,86]. In the HeLa cells, we found that constitutive expression of a dominant negative p53 mutant was capable of sensitizing the HeLa cells to HDAP to the same extent as HPV E6 [80]. This result demonstrated that p53 inactivation can sensitize cells to HDAP.

Interestingly, several reports provide evidence for direct effects of HSV on p53 levels. Like HPV E6, the ICP0 protein of HSV-1 was found to bind to and mediate the ubiquitination of p53 *in vitro* [87]. A similar ICP0-dependent ubiquitination was apparent in murine fibroblasts cotransfected with human p53 and ICP0 expression plasmids. However, the destabilization of p53 is not detected during an HSV infection. In fact, p53 was found to be phosphorylated and stabilized in human foreskin fibroblasts and primary mammary epithelial cells infected with HSV-1 [88] and [Nguyen and Blaho, unpublished results]. Furthermore, human embryonic lung cells infected with a replication defective mutant of HSV, which expresses ICP0, displayed p53 stabilization and p21 upregulation [89]. Therefore, the effects of ICP0 on p53 may be overridden by other HSV genes during a complete virus infection.

#### 2.4.2. Telomerase

The hTERT gene encodes the catalytic component of human telomerase [90–93]. Telomerase is the enzyme which replicates the ends of chromosomes (telomeres) [94]. Telomeres are typically shorter in adult somatic cells than those of embryonic and tumor origin [95–97]. Without telomerase, telomeres progressively shorten and when they reach a critical length, cells stop dividing or die through a process known as crisis [98]. Because HPV E6 is known to increase telomerase activity through upregulation of hTERT [99], we studied the effects of hTERT expression on sensitivity to HDAP. HeLa cells constitutively overexpressing hTERT, were as sensitive to HDAP as those cells expressing only HPV E6 [80]. Furthermore, primary mammary epithelial cells immortalized by hTERT expression are sensitive to HDAP, while primary mammary epithelial cells are resistant [Nguyen and Blaho, unpublished results]. Therefore, hTERT overexpression sensitizes cells to viral apoptosis. Contrary to HDAP, hTERT expression has been reported to decrease sensitivity to various other apoptotic stimuli, including oxidative stress, serum deprivation, dsDNA breaks, and cisplatinum [100–103]. Thus, HSV seems to be manipulating the apoptotic pathway in a unique way.

## 3. Conclusions

Apoptosis is a common cellular defense against pathogens. As a consequence, many successful pathogens possess an ability to modulate the apoptotic response. Over a decade of research has revealed much about the regulation of apoptosis during HSV infection. We know that apoptosis is initially triggered during the expression of viral immediate early genes, but this cell death signal is later squelched by the production of antiapoptotic viral factors. In this way, an HSV-infected cell walks a fine line of apoptotic balance during a productive infection, delaying cell death, presumably until the moment optimal for virus replication.

**Figure 1.**
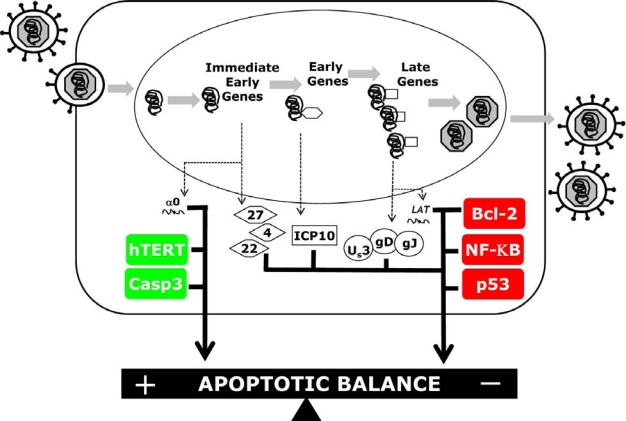
Viral and Cellular players in the HSV dependent apoptosis balancing act. Shown here is a current model of apoptotic modulation during an HSV infection. During the early stages of viral infection, immediate early viral gene expression triggers apoptosis. The cellular factors, hTERT and caspase 3, contribute to apoptosis induction. However, at later times post infection, early and late viral anti-apoptotic genes are produced, which block apoptosis from proceeding. Bcl-2, NF-κB, and p53 are cellular genes that contribute to the blocking of apoptosis during infection. Collectively, this sets up a delicate balance between the pro- and anti-apoptotic factors during a productive HSV infection.

Soon after the viral factors involved in apoptosis were elucidated, cell specific differences in sensitivity to HSV induced apoptosis were recognized. The identification of cellular proteins which mediated these cell type specificities followed (Figure 1). The cellular mediators of apoptosis Bcl-2 family members and caspases relay the signal from the initiation to execution of cell death during HSV infection. Exploiting the apoptotic proteins that are necessary for HDAP may clarify HDAP’s impact on viral pathogenesis. Less predictably, perhaps, were the findings that at least two genes involved in tumorigenesis, p53 and hTERT, influence the ability of the infected cell to respond to viral apoptotic triggers. At this time, the molecular mechanisms whereby these genes confer their effects is still unclear. What is clear is that the regulation of apoptosis within an HSV infected cell is much more complex that originally anticipated. We have yet to learn how many more cellular players may impact the HSV dependent apoptosis balancing act.
